# Pulmonary arterial hypertension caused by congenital extrahepatic portocaval shunt: a case report

**DOI:** 10.1186/s12872-019-1124-1

**Published:** 2019-06-13

**Authors:** Kai-yang Lin, Hui Chen, Ling Yu

**Affiliations:** Department of Cardiology, Fujian Provincial Hospital, Fujian Medical University, Fujian Cardiovascular Institute, Fuzhou, 350001 China

**Keywords:** Abernethy malformation, Congenital extrahepatic portocaval shunt, Pulmonary arterial hypertension

## Abstract

**Background:**

Congenital extrahepatic portocaval shunt (CEPS), also known as Abernethy malformation, is an extremely rare anomaly of the splanchnic venous system, especially when accompanied by pulmonary arterial hypertension.

**Case presentation:**

We report a case of a 15-year-old female who was diagnosed with CEPS (Abernethy type Ib) accompanied by pulmonary arterial hypertension. This case was incidentally identified during abdominal ultrasound examination and confirmed by mesenteric and splenic arteriography. During more than 4 years of follow-up, after receiving sildenafil (80 mg/day), the patient’s condition improved in the first year after discharge. However, one year later, the patient’s conditions start to deteriorate.

**Conclusion:**

This article presents a rare case of Abernethy malformation accompanied by pulmonary arterial hypertension, which can be diagnosed by using abdominal ultrasonography, portal vein computed tomography angiography or mesenteric and splenic arteriography. This malformation had limited treatment and poor prognosis.

## Case report

Congenital extrahepatic portocaval shunt (CEPS) is a congenial anomaly observed predominantly in females in which splanchnic blood bypasses the liver and drains directly into the inferior vena cava (IVC) [1]. Since the first description by Abernethy in 1793 [2], fewer than 100 cases of CEPS have been reported, and only a few cases accompanied by pulmonary hypertension have been described [3–7]. Here, we present a case of CEPS with pulmonary arterial hypertension in a female adolescent patient.

A 15-year-old female was admitted to our department with shortness of breath. One year prior to admission, she started to feel shortness of breath when walking fast. One month before admission, she suffered from nasal congestion, rhinorrhoea, and cough. On admission, the patient had severe dyspnoea (New York Heart Association, NYHA class IV). In addition, physical examination showed a systolic murmur heard loudest in the second left intercostal space, peripheral oedema, and splenomegaly.

**Fig. 1:**
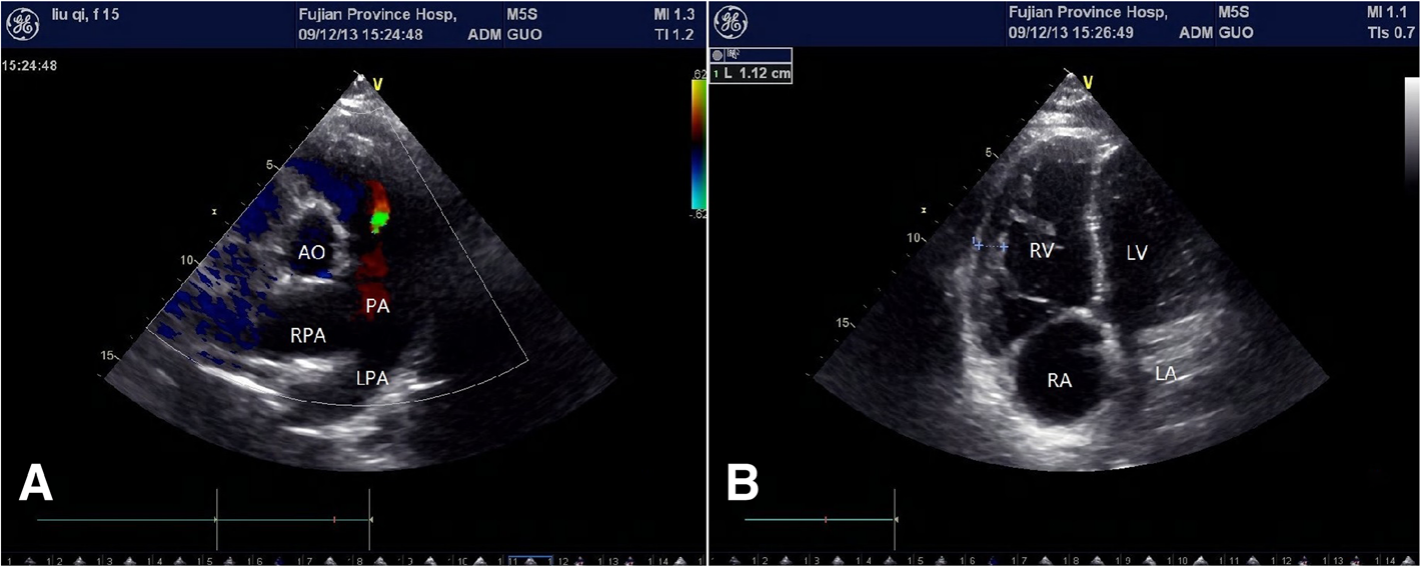
Echocardiography shows an enlargement of the PA and its branches (**a**). Both RA and RV are significantly dilated, and the RV wall thickness is increased (**b**). PA: pulmonary artery; RPA: right pulmonary artery; LPA: left pulmonary artery; AO: aorta; RA: right atrium; RV: right ventricle; LA: left atrium; LV: left ventricle

Laboratory tests showed a decrease in platelet count (55 × 10^9/L; reference range, 125–350) and serum albumin (30 g/L; reference range, 40–55) and an increasement in bilirubin (12.3 μmol/L; reference range, 0.0–5.1). Electrocardiogram (ECG) revealed right axis deviation, right ventricular hypertrophy and T-wave changes in precordial leads. The echocardiography also showed enlargement of the pulmonary artery and its branches (Fig. 1a). Additionally, both the right atrium (RA) and right ventricle (RV) were significantly dilated (the inner diameters of RA and RV were 4.9 cm and 4.1 cm, respectively, measured in the four chamber view), and the RV wall thickness was increased (0.99–1.12 cm) (Fig. 1b). The estimated RV systolic pressure (RVSP) was 97 mmHg, which calculated from the maximal velocity of tricuspid regurgitation,.Right catheterization was performed to further evaluate the pulmonary arterial pressure, demonstrating right atrial pressure (RAP) of 17/4/9 mmHg, right ventricular pressure (RVP) of 104/40/62 mmHg, pulmonary capillary wedge pressure (PCWP) of 11/2/7 mmHg, pulmonary arterial pressure (PAP) of 105/43/64 mmHg, pulmonary vascular resistance (PVR) of 12.63 woodU and a cardiac index (CI) of 2.44 L/min/m^2^.

**Fig. 2:**
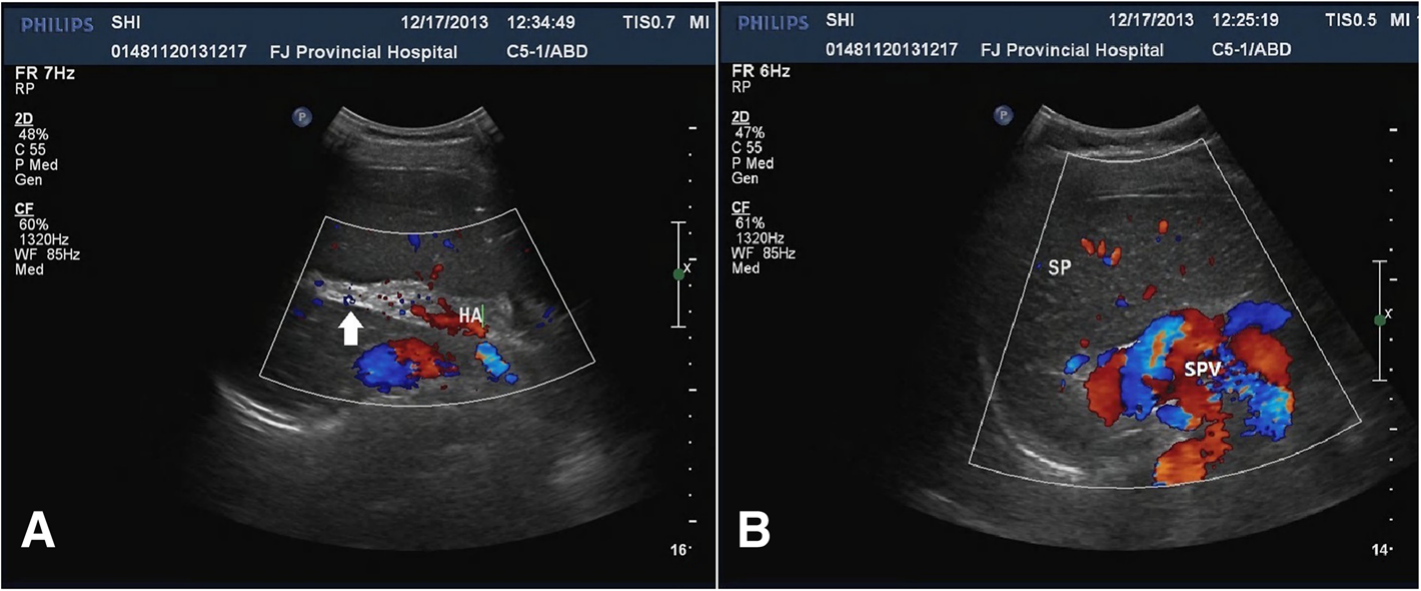
Abdominal ultrasonography shows that the intrahepatic PV system is absent (arrows) (**a**), and the SPV is tortuous and dilated (**b**). PV: portal vein; HA: hepatic artery; SP: spleen; SPV: splenic vein

**Fig. 3:**
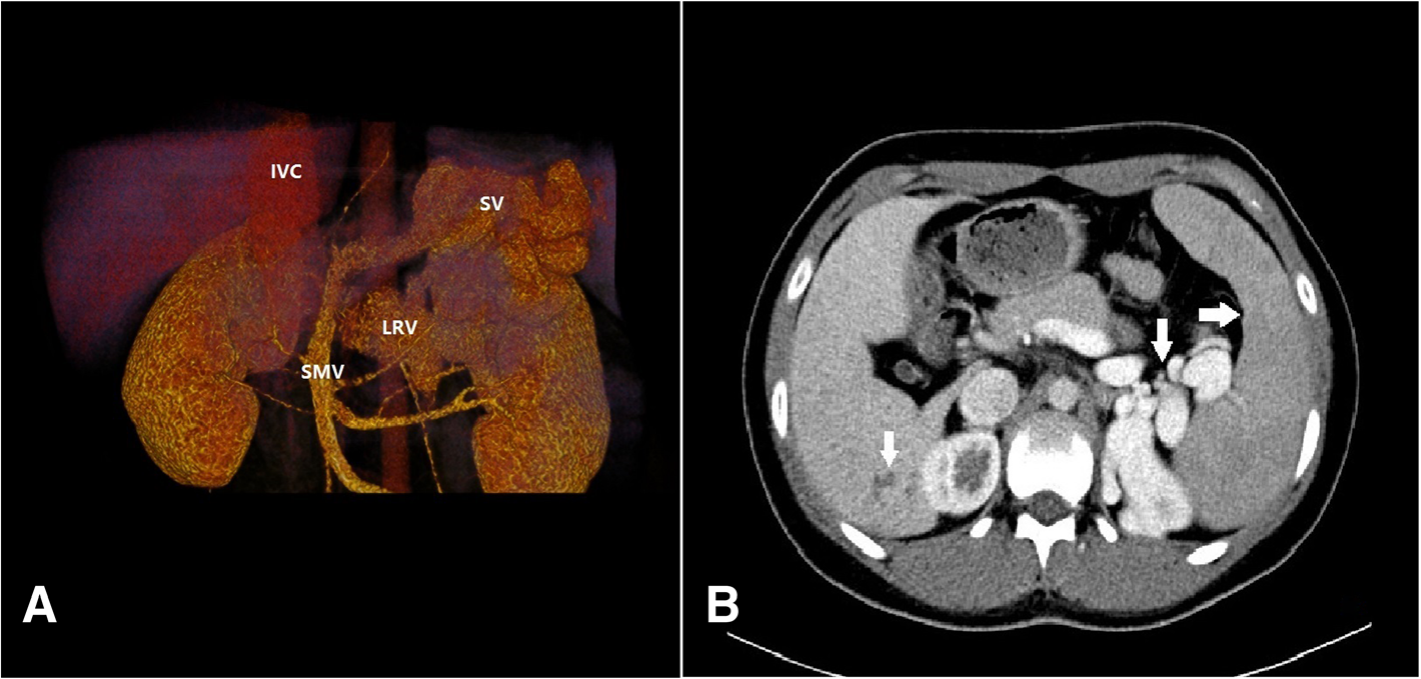
PV-CTA shows that the trunk and branches of PV are absent, the SV is widened, with the SMV joining the LRV and then draining directly into the IVC without passing through the liver (**a**). CTA also reveals the enlargement of the spleen and splenic veins (arrows) and heterogeneous density lesions in the liver nodules (arrows) (**b**). PV: portal vein; CTA: computed tomography angiography; SV: splenic vein; SMV: superior mesenteric vein; LRV: left renal vein; IVC: inferior vena cava

**Fig. 4:**
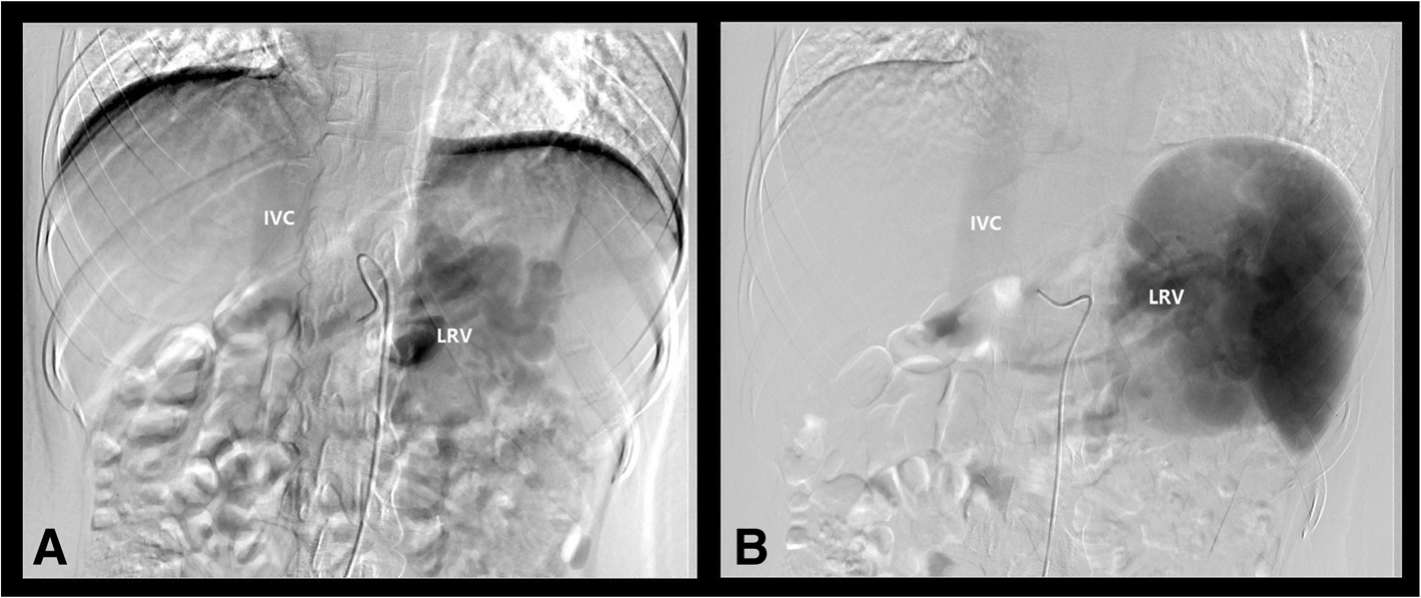
Mesenteric and splenic arteriography shows that the portal vein was absent, and the shunt between splenic and renal venous supply and the LRV and IVC can be seen in the delayed phase (**a**, **b**). LRV: left renal vein; IVC: inferior vena cava

Incidentally, abdominal ultrasonography revealed the absence of an intrahepatic portal vein system and a tortuous and dilated splenic vein (Fig. 2a, b). Further evaluation using computed tomography angiography (CTA) of the portal vein demonstrated the absence of a portal vein and dilation of the splenic vein (SV), while the superior mesenteric vein (SMV) was connected to the left renal vein (LRV) through a large number of collateral vessels that drained directly into the IVC bypass the liver (Fig. 3a). CT also revealed heterogeneous density lesions in the liver nodules, tortuous and dilated left renal and splenic veins, and enlargement of the spleen (Fig. 3b). Mesenteric and splenic arteriography were performed to confirm the diagnosis. In the delayed phase, the portal vein was absent, but the shunt between the splenic and renal venous supply and the left renal vein and inferior vena cava was observed (Fig. 4a, b).

**Fig. 5:**
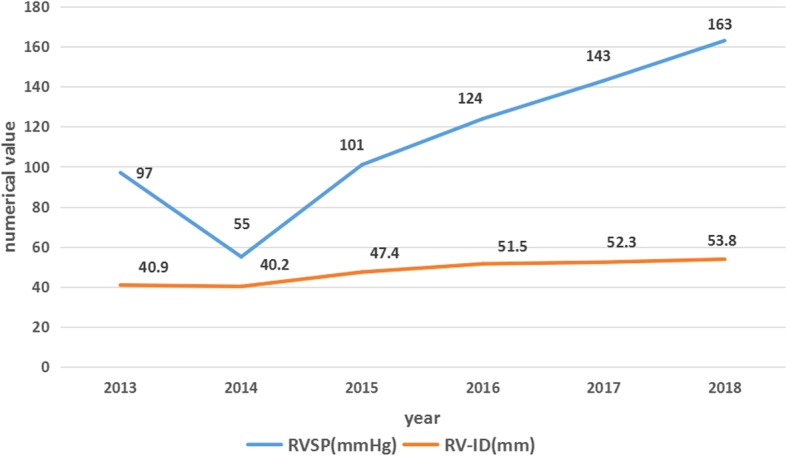
Changes in important parameters in echocardiography. RVSP: right ventricle systolic pressure; RA: right atrium; RV-ID: right ventricle inner diameter

Based on the findings described above, we established a diagnosis of Abernethy malformation (type Ib) with pulmonary arterial hypertension. The patient refused surgical treatment and received sildenafil (80 mg/day). Her conditions were improved, and she was discharged on day 12. After discharge, the patient was subject to follow-up clinical visits. During the first year after discharge, the patient’s symptoms (such as shortness of breath and fatigue) were improved significantly. With continuous use of sildenafil, however, the patient’s symptoms (such as shortness of breath and fatigue) were aggravated, and the related parameters of echocardiography were also worsening at one year later (Fig. 5).

## Discussion and conclusions

CEPS is a rare congenital anomaly caused by the abnormal embryonic development of the portal vein. CEPS is also known as the Abernethy malformation because it was first reported by John Abernethy in 1793 in an autopsy of a 10-month-old infant who died of unknown reasons [2]. In 1994, Morgan and Superina [8] classified CEPS into two types based on the presence or absence of an intrahepatic portal venous supply. Type I is characterized by the absence of the intrahepatic PV with complete diversion of portal blood into system veins (end-to-side shunts). Type I is further subdivided into two subtypes: (i) separate drainage of the superior mesenteric and splenic veins into the IVC, iliac veins, or renal veins (Ia); and (ii) superior mesenteric and splenic veins are joined to form a short extrahepatic PV that drains into the IVC (Ib). Type II is characterized by the presence of a hypoplastic portal vein with portal blood diversion into the vena cava through side-to-side, extrahepatic communication.

Under normal conditions, portal venous blood carrying nutrients drains into the hepatic vein through the liver sinusoidal system and then returns to the inferior vena cava. In CEPS, as a lack of hepatic portal vein perfusion or hypoperfusion, nutrients and active substances absorbed by the gastrointestinal tract enter systemic circulation, directly bypassing the liver and leading to a series of pathophysiological changes and clinical syndromes. The clinical presentations of CEPS may vary and depend on associated conditions: (1) liver dysfunction and hepatic neoplasms, which may be benign (focal nodular hyperplasia and nodular regenerative hyperplasia) or malignant (liver cancer), may be due to the lack of perfusion and nutrient delivery to the liver [9–11]. (2) Congenital hepatic shunts can also present together with hepatic encephalopathy, hepatopulmonary syndrome [12], and hypoglycaemia [13]. (3) Increased pressure of the splenic vein and superior mesenteric vein system with complete or partial occlusion of the portal vein can lead to hypersplenism and haemorrhage of the digestive tract. (4) Congenital extrahepatic portosystemic shunts are frequently associated with other anomalies, including congenital heart disease, polysplenia, biliary atresia, malrotation, duodenal atresia, annular pancreas, situs inversus, renal tract anomalies, and skeletal anomalies [14]. Type I Abernethy malformation frequently occurs in children and females and is often associated with other malformations and complications, such as congenital heart disease, polysplenia, biliary atresia, hepatic encephalopathy, and hepatopulmonary syndrome. Type II Abernethy malformation is more common in males and is rarely associated with the above malformations [14].

It is difficult to diagnose Abernethy malformation based on the clinical presentations only. The image characteristics of this disease are portal venous malformation, in which the trunk and branches of the portal vein are absent or atretic. Angiography is the gold standard for the diagnosis of CEPS but is not the first imaging study commonly employed because it is an invasive examination that may not identify other visceral lesions at the same time [15]. Non-invasive cross-sectional imaging techniques, such as ultrasound, CT or MRI can be used as the first choice for diagnosis, which can also show the shunt and intrahepatic portal vein branches.

To our knowledge, CEPS is rarely reported as a case associated with pulmonary arterial hypertension. In our case, we incidentally found portal atresia by abdominal ultrasound examination, which was then confirmed by portal vein CTA and mesenteric and splenic arteriography. Finally, we diagnosed the patient with Abernethy malformation (type Ib) according to the imaging findings, which showed the absence of a portal vein and the joining of the superior mesenteric veins with the splenic veins.

Pulmonary hypertension develops in approximately 2% of patients with portal hypertension [16], which is called portopulmonary hypertension (PPHT). The pathogenesis of pulmonary hypertension caused by CEPS may be interpreted as PPHT, which includes the following [17]. First, this condition is caused by an imbalance between vasoconstrictors and vasodilators promoting pulmonary vasoconstriction. Second, the hyperdynamic circulation contributes to the development of pulmonary hypertension. Third, the increased pulmonary blood flow may produce excessive shear stress that leads to endothelial injury and dysfunction with vasoconstriction and progressive vascular remodelling. Finally, the vasoconstrictive and proliferative substances that bypass the liver without metabolism, reach the pulmonary vascular bed directly and act on pulmonary vasculature may lead to pulmonary hypertension.

Unfortunately, there is still no unified experience or consensus in the treatment of Abernethy malformation [14]. The treatment plan is dependent on the type of shunt and the individual conditions. Treatment for CEPS usually includes medications and surgery. For patients with type I CEPS who develop severe complications, such as encephalopathy, hepatopulmonary syndrome, pulmonary hypertension or neoplasms, liver transplantation and portal vein reconstruction appear to be the best treatment options. Patients with type II CEPS with portal hypertension and hepatic encephalopathy can benefit from early shunt occlusion surgery [14, 18, 19].

## Data Availability

All data generated or analysed during the study are included in the published article.

## Abbreviations

CEPS: Congenital extrahepatic portocaval shunt
IVC: Inferior vena cava
PV: Portal vein
SPV: Splenic vein
